# Biological Responses of Nile tilapia *Oreochromis niloticus* as Influenced by Dietary Florfenicol

**DOI:** 10.3390/toxics10100571

**Published:** 2022-09-29

**Authors:** Avishek Bardhan, Thangapalam J. Abraham, Ratnapriya Das, Prasanna K. Patil

**Affiliations:** 1 Department of Aquatic Animal Health, West Bengal University of Animal and Fishery Sciences, Chakgaria, Kolkata 700094, India; 2 Aquatic Animal Health and Environment Division, ICAR—Central Institute of Brackishwater Aquaculture, Chennai 600028, India

**Keywords:** aquaculture, antibiotics, biomarkers, hematological responses, histopathology, erythrocytes

## Abstract

Antibiotics are used in the treatment of bacterial diseases in commercial aquaculture. In this study, we the biological responses of *Oreochromis niloticus* juveniles upon dietary florfenicol (FFC) administration at 15 mg (1×) and 45 mg kg biomass^−1^ day^−1^ (3×) for 10 days in terms of feed intake, survival, biomass, hematological, erythro-morphological, serum biochemical, and histopathological aberrations as compared with controls. FFC caused a dose-dependent reduction in feed intake, survival, and biomass, with marked variations in hematology, hematological indices, and erythrocytic cellular and nuclear abnormalities, suggesting its apparent cytotoxic and nucleotoxic effects. The serum biomarkers increased significantly in a dose-dependent manner, except for calcium and chloride, which decreased significantly. The therapeutic dose (1×) group exhibited marked histopathological aberrations, such as renal tubular epithelial degeneration and a widened lumen in the kidney, as well as glycogen-type vacuolation and cytoplasmic degeneration in the liver during the dosing period. The extent of kidney and liver tissue damage was more prominent in the 3× group. The 1× serum biomarker levels became normal, with the exception of alkaline phosphatase, within 3 weeks of suspension of dosing. The recovery of the measured parameters and histopathological and erythro-morphological changes suggested that the therapeutic dietary biological responses induced by FFC are reversible and safe for *O. niloticus*.

## 1. Introduction

In the aquaculture industry, diseases are considered a major constraint. Feeding infected fish with antibiotic-medicated food is a general practice, with the aim of mitigating bacterial diseases [1]. Florfenicol (FFC) is a synthetic, fluorinated analog of thiamphenicol [2], specifically developed for use in veterinary medicine with registered uses in fish and other food-producing animals. It has been conditionally approved for use in aquaculture and is the most recent antibiotic to be approved after sulfadimethoxine/ormetoprim, oxytetracycline dihydrate, and sulfamerazine [1]. In food animals, FFC has shown promise as a replacement for sulphonamides and tetracyclines, which have been associated with toxicity and residue concerns [3]. In fish, FFC is administered in medicated feed for 10 consecutive days at doses of 10–15 mg kg biomass^−1^ day^−1^ [1]. Globally, tilapias are the most widely grown type of intensively reared farmed fish, second only to carp in importance. Nile tilapia *Oreochromis niloticus* contributed 80% to the worldwide production of tilapias [4]. The demonstration of the potential effects of dietary FFC in tilapias has been limited to safety studies [5,6] and to controlling susceptible bacterial pathogens [7]. Florfenicol is highly lipophilic and can cross anatomic barriers, supporting its pathogen-efficacy [1]. This particular characteristic of FFC can have a detrimental effect on piscine blood cells [6]. Several works are available reporting the antibiotic cytotoxicity and alterations in hematological parameters of *Labeo rohita* [8] and *Cirrhinus mrigala* [9].

The data concerning hematological responses in fish after antibiotic treatment are ambiguous. The results obtained by various authors probably depend on the antibiotic dosage and the sensitivity of various fish species [10,11,12]. Antibiotics are known to distress the morphology of fish blood cells. Our earlier work on erythro-morphological alterations during extended FFC-feeding [6] suggested probable anaemic conditions in *O. niloticus* juveniles. The cytotoxic effects of FFC are well-known [13]. However, biological responses to dietary FFC, along with the associated hematological and histoarchitectural alterations, have been vaguely studied and interrelated in relation to commercially important aquaculture fish species in tropical Indian conditions with ambient temperatures above 25 °C. Therefore, this study was conducted to assess such responses in juvenile *O. niloticus* upon dietary FFC administration at target doses of zero (control), one, and three times the maximum therapeutic dose of 15 mg kg biomass^−1^ day^−1^ for 10 consecutive days (therapeutic duration). Since drug metabolism and excretion are primarily concerned with the liver and kidneys, respectively, we targeted these two organs for histopathological alterations.

## 2. Materials and Methods

### 2.1. Experimental Fish and Design

Healthy Nile tilapia *Oreochromis niloticus* juveniles (30.74 ± 0.80 g; 12.72 ± 0.21 cm) were procured from a grow-out farm located in Sonarpur (Lat. 22°27′50.2158″ N; Long. 88°23′7.4004″ E), West Bengal, India, and transported to the laboratory in oxygen-filled plastic bags. The juveniles were acclimatized for 15 days with constant aeration and fed with a commercial floating pelleted feed of 2 mm in diameter (Aquaxcel, Cargill, India, 8% fat, 42% protein, 5% fiber, and 11% moisture) thrice daily at 2% body weight [6]. The juveniles with no infections were randomly collected from the stock tanks, relocated into 9 circular fiberglass reinforced plastic (FRP) tanks with 40 fish each, and acclimatized for 7 days (pre-dosing period). The fish were allotted into 3 groups, viz., group 1: 0× control, group 2: 1× FFC-dosing (15 mg kg biomass^−1^ day^−1^) and group 3: 3× FFC-dosing (45 mg kg biomass^−1^ day^−1^), in triplicates. About 50% of the water was flushed out and replaced in three interval days to remove wastes and feces. The fish were accustomed to the laboratory conditions for 3 weeks with continuous aeration. The water quality parameters were monitored bi-weekly and maintained at optimal levels (temperature: 22.00 °C–30.00 °C, pH: 7.80–8.60, dissolved oxygen: 4.90–5.20 mg/L, ammonia: 0.002–0.008 mg/L, nitrite: 0.14–0.53 mg/L and nitrate: 0.13–0.55 mg/L) throughout the experimental period.

### 2.2. Florfenicol Diet Preparation and Dose Administration

The highest therapeutic dose of FFC is 15 mg kg biomass^−1^ day^−1^ for 10 consecutive days [1]. The inclusion rate for the FFC (Tokyo Chemical Industry, CAS RN: 73231-34-2; Product Number: F0811-5g) was calculated to deliver an approximate dosage of 0–45 mg FFC kg biomass^−1^ day^−1^ for 10 consecutive days. The feed top-coating of FFC using vegetable oil as a binder was carried out as described in a previous paper [6]. The 38-day study included 7 days of acclimation, 10 days of FFC dosing (FD), and 21 days of post-FFC dosing (PFD) periods. During the pre- and post-dosing periods, the fish groups were fed a control diet. During the dosing period, control and FFC diets were administered to the respective groups thrice daily. The daily ration, 2% BW, was allocated into 3 equivalent portions. Feed remaining in tank 1 h after each feeding period was siphoned out into a pre-weighed container, dried overnight, pooled tank-wise on daily basis, and weighed. Feeding activity was visually assessed thrice daily. Observations of feed consumption, behavioral changes, external changes, and mortality were recorded daily. The feed ration was adjusted with the biomass accrual and mortalities. Behaviors such as swimming to the surface during feeding, aggressive feeding, and distribution throughout the water column were considered normal. External changes such as pigmentation and gross lesions were also observed during feeding.

### 2.3. Blood Sampling

Blood sampling to assess serum biomarkers was carried out on days 0 and 10 FD and days 1, 7, 14, and 21 PFD for each group. Before the blood collection, three fish from each tank were anesthetized using clove oil (20 μL L^−1^). The blood was collected via a caudal vein puncture [14] using a 2 mL sterile plastic syringe. Instantly, 2 drops of non-heparinized blood were taken on microscopic slides, followed by blood smear preparation. The left-out non-heparinized blood in the syringe was allowed to clot and was then incubated at 4 °C overnight. The serum was collected via centrifugation at 1000× *g* for 15 min, transferred to Eppendorf tubes, and stored at −20 °C for further analysis. Blood samples for hematology were taken and observations of blood morphological alterations were made on days 0 and 10 FD and day 21 PFD. The blood was drawn as above and instantaneously transferred to anticoagulant-coated vials for further analysis.

### 2.4. Serum Biomarkers

The serum biomarkers of stress (glucose), liver function (alanine aminotransferase (ALT), aspartate aminotransferase (AST), and alkaline phosphatase (ALP)), kidney function (creatinine), and ionic balance (calcium and chloride) were determined using commercial kits in a Photometer (Model: 5010 v5+, Robert Riele KG, Berlin, Germany) (Table 1).

### 2.5. Hematology and Hematological Indices

Evaluation of hematological parameters and indices was performed as per the standard protocol [21] with slight modifications. The enumeration of total erythrocyte counts (TECs), total leukocyte counts (TLCs), and thrombocyte counts (TCs) of fish blood was conducted in Neubauer’s counting chamber using Hayem’s fluid, Shaw’s white blood cell diluting fluid, and Rees–Ecker diluting fluid, respectively. Differential leukocyte counts, such as lymphocyte counts (LCs) and monocyte counts (MCs), were enumerated through the analysis of stained blood smears, in which the numbers of various types of leukocytes were counted/100 cells in several fields of the smear. The hemoglobin (Hb) levels were estimated via the acid-hematin method using Sahli’s hemocytometer. The hematocrit value (Ht) was determined via micro-hematocrit centrifugation. The hematological indices (mean corpuscular volume (MCV), mean corpuscular hemoglobin (MCH), and mean corpuscular hemoglobin concentration (MCHC)) were computed based on the Hb, Ht, and TEC results.

### 2.6. Erythrocyte Morphological Characters

Following the blood smear preparations, the slides were stained with 5% Giemsa and 0.2% safranin separately, then were dried and observed under a microscope with a 10× (ocular) and 100× (objective) lens using immersion oil as described in a prior paper [6]. Photomicrography was accomplished using an advanced trinocular research microscope (Olympus, Tokyo, Japan, Model: BX51) and using an SCO-LUX 16 MP camera. ToupTek ToupView software (Version x64, 4.11) was used for image capture and processing. Erythrocytes of FFC-dosed *O. niloticus* with morphological abnormalities at the cellular and nuclear levels, such as vacuolation, cell membrane deformities, cell membrane rupture, damaged erythrocytes, and other anomalies, were documented as described in earlier reports [22,23].

### 2.7. Histopathology

On days 0 and 10 FD and day 21 PFD, the kidney and liver tissues of *O. niloticus* juveniles were collected and fixed in Bouin’s solution for 24 h. The tissue processing, embedding, sectioning (5 µm), and hematoxylin and eosin staining have been described previously [14]. Image capture and processing for photomicrography were accomplished as described above. After the image capture step, the major histopathological adversities in these two organs were assessed in comparison with the normal architecture and were transformed into qualitative scores on an ordinal scale [24].

### 2.8. Statistical Analyses

The data were expressed as a mean ± standard deviation. To know the significance of differences in each of the parameters and indices on the scheduled day among treatments and each of the specific treatments among days, one-way ANOVA, followed by the Tukey HSD post hoc test for the comparison of means, was carried out. Significant differences in the histopathological scores of kidney and liver tissues on scheduled days and among treatments were evaluated using the Kruskal–Wallis non-parametric test. All the statistical analyses were implemented using Statistical Package for Social Sciences (IBM-SPSS) Version: 22.0, considering a probability level of *p* < 0.05.

## 3. Results

### 3.1. Feed Intake, Survival, and Biomass

**Figure 1:**
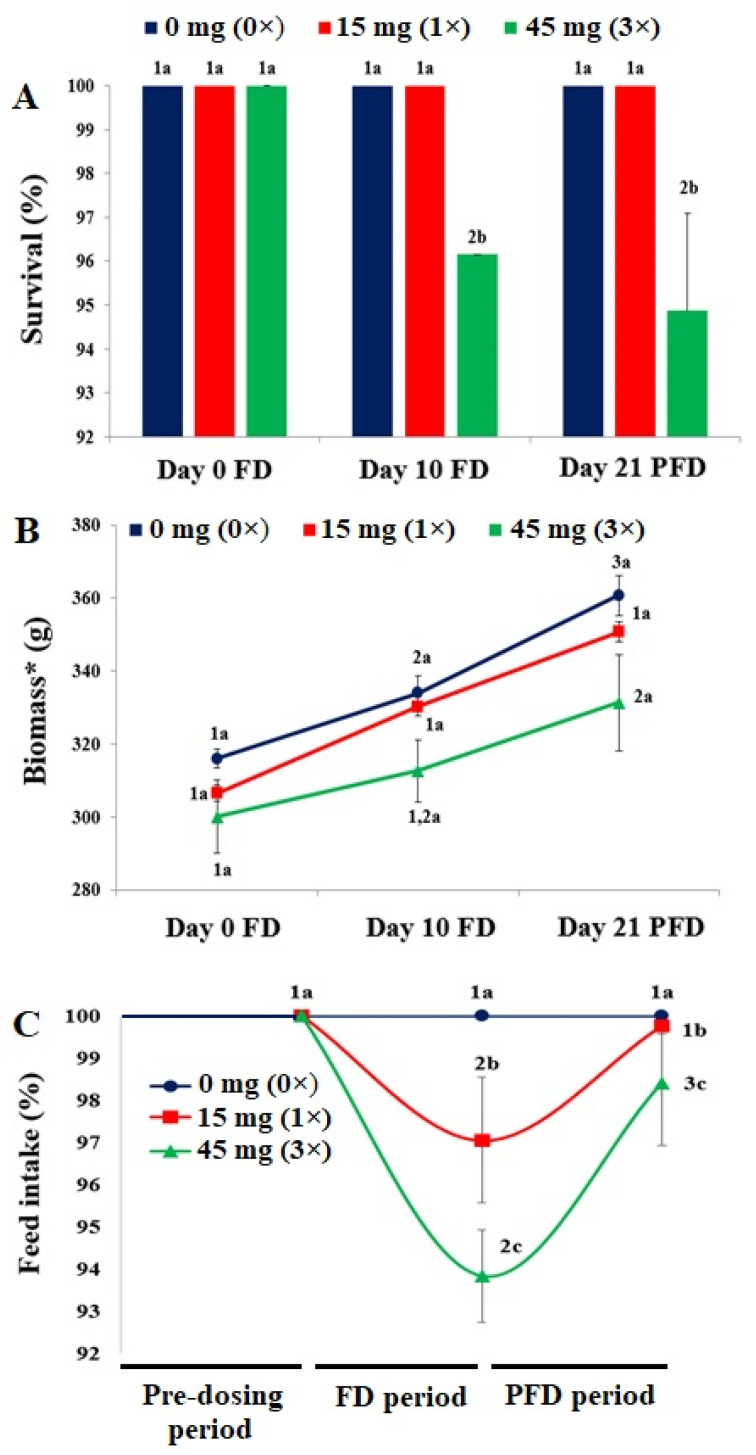
Effects of florfenicol (FFC) dosing at 0–3 times the therapeutic dose of 15 mg kg biomass^−1^ day^−1^ for 10 consecutive days on (**A**) survival, (**B**) biomass, and (**C**) feed intake of *Oreochromis niloticus* juveniles during the experimental period. Pre-dosing period: 0–7 days; FFC-dosing (FD) period: 8–17 days; post-FFC-dosing (PFD) period: 17–38 days. * Biomasses of 10 fish from each of the triplicate tanks. a–c: bars and line markers sharing a common alphabet for a particular day or dosing period differed insignificantly (*p* > 0.05). 1–3: bars and line markers sharing a common numeral for a particular treatment (dose) differed insignificantly (*p* > 0.05).

The control fish displayed active feeding throughout the experiment. No abnormal behavioral changes were observed in the dosing groups. The 1× group consumed 97% of the feed offered during the FD tenure, which steadily increased during the PFD period. The 3× group showed a significant reduction in feed intake during the FD regimen, which significantly increased (*p* < 0.05) upon withdrawal (Figure 1C). The control and 1× groups exhibited 100% survival, whereas the 3× group exhibited reduced survival on day 10 FD and day 21 PFD (Figure 1A). The biomass of FFC-dosed *O. niloticus* increased in all groups, with the highest observed in the control group, followed by the 1× group (Figure 1B). The behavioral abnormalities in juveniles upon FFC administration were visually evaluated and displayed in Table 2. These abnormalities were considered to be mild. The internal organs of *O. niloticus* showed no abnormal pigmentation or alterations, except for the 3× group, which demonstrated an enlarged liver upon necropsy of a freshly dead fish. The liver enlargement persisted until day 21 of PFD.

### 3.2. Serum Biomarkers

**Figure 2:**
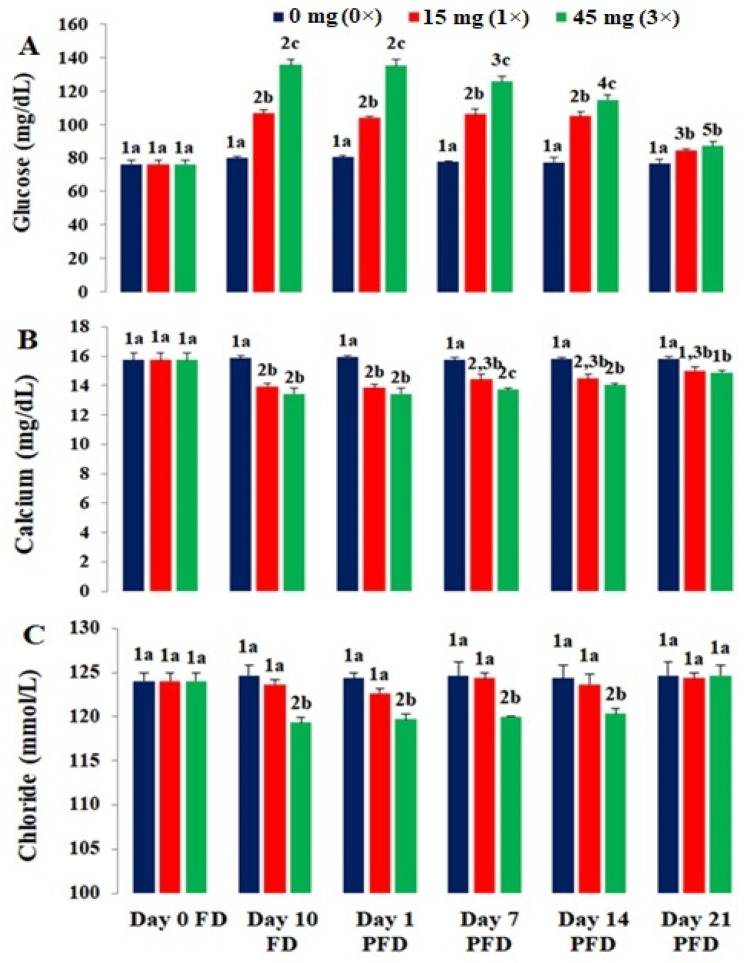
Effects of florfenicol (FFC) dosing at 0–3 times the therapeutic dose of 15 mg kg biomass^−1^ day^−1^ for 10 consecutive days on the serum (**A**) glucose, (**B**) calcium, and (**C**) chloride levels of *Oreochromis niloticus* juveniles. a–c: bars sharing a common alphabet for a particular day differed insignificantly (*p* > 0.05). 1–5: bars sharing a common numerical for a particular treatment (dose) differed insignificantly (*p* > 0.05).

**Figure 3:**
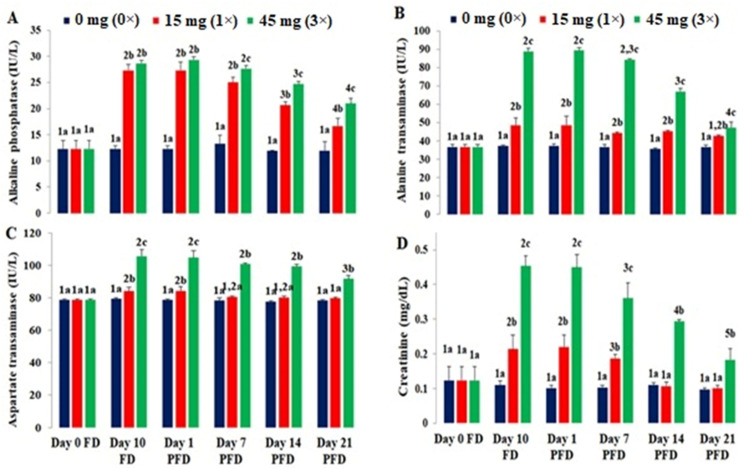
Effects of florfenicol (FFC) dosing at 0–3 times the therapeutic dose of 15 mg kg biomass^−1^ day^−1^ for 10 consecutive days on the serum (**A**) alkaline phosphatase (ALP), (**B**) alanine transaminase (ALT), (**C**) aspartate transaminase (AST), and (**D**) creatinine levels of *Oreochromis niloticus* juveniles. a–c: bars sharing a common alphabet for a particular day differed insignificantly (*p* > 0.05). 1–5: bars sharing a common numerical for a particular treatment (dose) differed insignificantly (*p* > 0.05).

The results regarding the serum biomarkers of the control and FFC-dosed *O. niloticus* are presented in Figure 2A–C and Figure 3A–D. All the dosing groups showed a significant increment in glucose levels (p < 0.05) on day 10 FD (Figure 2A), followed by a significant reduction (*p* < 0.05) on day 21 PFD. The recorded decline in glucose levels of the 1× group on days 1, 7, and 14 PFD was insignificant (*p* > 0.05), but it was significant on day 21 PFD (p < 0.05). The glucose levels of both groups were comparable on day 21 PFD but significantly higher than the control. A significant decline in calcium levels was observed in the 1× and 3× groups up to day 1 PFD (*p* < 0.05) and these levels increased thereafter (Figure 2B). These levels were recovered on day 21 of PFD. The chloride levels of the 1× group were reduced but insignificantly until day 1 PFD (*p* > 0.05), whereas those of the 3× group decreased significantly (*p* < 0.05) on day 10 FD (Figure 2C). The difference in chloride levels between the 1× and control groups was insignificant (*p* > 0.05). The ALP levels of the 1× and 3× groups were significantly higher than the controls on day 10 FD (*p* < 0.05), followed by a significant decrease (*p* < 0.05) from day 14 PFD. A significant difference (*p* < 0.05) persisted in the ALP levels of the 1× and 3× groups on day 21 of PFD (Figure 3A). On day 10 FD, the ALT levels of the 1× group were significantly higher than those of the controls (*p* < 0.05). Although the ALT levels recovered slightly in the 1× group, they were still significantly higher than those of the control gorup on day 21 PFD. A similar trend was noted in the 3× group (Figure 3B). In the 1× and 3× groups, significantly high AST levels (*p* < 0.05) were noted on day 10 FD, which were reduced with the termination of dosing. The AST levels of the 3× group were significantly higher (*p* < 0.05) than those in the 1× group. Their levels in the 1× group returned almost to normal on day 7 PFD. These high levels persevered in the 3× group and did not recover even on day 21 PFD (Figure 3C). The creatinine levels of the 1× and 3× groups reached a peak on day 10 FD and started to drop by day 7 PFD. The creatinine levels of the 1× and 3× groups on day 10 FD and days 1 and 7 PFD differed significantly (*p* < 0.05). These level in the 1× group recovered on day 14 PFD but were still higher in the 3× group (Figure 3D).

### 3.3. Hematology and Hematological Indices

The results concerning the hematology and hematological indices of the control and FFC-dosed *O. niloticus* are presented in Table 3. On day 10 FD, a significant reduction in TECs was recorded in the 1× group (*p* < 0.05), which then increased significantly on day 21 PFD. The TECs of the 3× group followed a similar trend. The difference in the TECs of the 1× and 3× groups on day 10 FD was insignificant (*p* > 0.05). The TCs of the 1× and 3× groups exhibited a significant increase on day 10 FD (*p* < 0.05). Subsequently, the counts abated significantly on day 21 PFD (*p* < 0.05). The TCs enumerated on day 21 PFD were essentially identical to those of the control group. The difference in the TCs was significant on day 10 FD (*p* < 0.05) and insignificant on day 21 PFD (*p* > 0.05). The TLCs of the 1× and 3× groups exhibited a significant hike on day 10 FD (*p* < 0.05). Upon the cessation of dosing, the TLCs decreased significantly (*p* > 0.05) in the 3× group on day 21 of PFD. The TLCs of the 1× and 3× groups were insignificantly different on day 21 PFD (*p* > 0.05). In the 1× and 3× groups, the LCs were significantly increased on day 10 FD (*p* < 0.05) and were subsequently found to be significantly curtailed on day 21 PFD (*p* < 0.05). The 1× group MCs showed a significant reduction on day 10 FD (*p* < 0.05). Upon the termination of dosing, the MCs increased significantly on day 21 PFD (*p* < 0.05). A similar trend was observed in the MCs of the 3× group.

The Hb levels of the 1× group were significantly reduced on day 10 FD, followed by a significant increase (*p* < 0.05) on day 21 PFD. Similarly, the Hb levels in the 3× group abated on day 10 FD and recovered insignificantly on day 21 PFD (*p* > 0.05). In the 1× group, the Ht values decreased insignificantly on day 10 FD (*p* > 0.05) and increased insignificantly on day 21 PFD. Similarly, the Ht values of the 3× group decreased significantly on day 10 FD (*p* < 0.05). On day 21 of PFD, the values increased insignificantly (*p* > 0.05). The MCV values in the 1× group significantly increased on day 10 FD and decreased significantly on day 21 PFD (*p* < 0.05). The 3× group also exhibited significantly high MCV values on day 10 FD (*p* < 0.05), which were consequently reduced on day 21 PFD. In the 1× group, the MCH values increased insignificantly on day 10 FD and then declined insignificantly on day 21 PFD (*p* > 0.05). The values in the 3× group increased significantly on day 10 FD (*p* < 0.05), followed by a significant decrease on day 21 PFD. The 1× and 3× groups showed a significant reduction in MCHC values on day 10 FD (*p* < 0.05). On day 21 of PFD, the MCHC values significantly increased (*p* < 0.05). The MCHC values of both groups were insignificantly different on day 10 FD, as well as on day 21 PFD (*p* > 0.05).

### 3.4. Blood Cell Morphological Alterations

**Figure 4:**
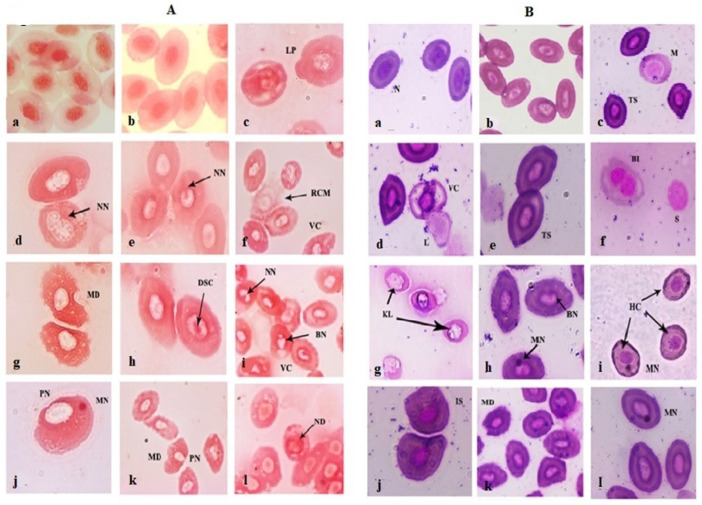
Effects of florfenicol dosing at 15 (1×) and 45 mg kg biomass^−1^ day^−1^ (3×) for 10 consecutive days on the erythrocyte morphological characteristics of *Oreochromis niloticus* juveniles on day 10 FD (1×: c–e; 3×: f–h) and day 21 PFD (1×: i,j; 3×: k,l) and compared with the control group (a,b). N: normal erythrocyte; LP: lobopodial extension; M: monocyte; TS: tear-drop-shaped erythrocyte; NN: notched nucleus; ND: nuclear deformity; RCM: ruptured cell membrane; VC: vacuolation; L: lymphocyte; KL: karyolysis; BI: bi-nucleated erythrocyte; S: smudge cell; MD: membrane deformity; DSC: darkly stained chromatin; BN: blebbed nucleus; MN: micronucleus; HC: hypochromic cell; PN: peripheral nucleus; IS: irregular-shaped erythrocyte. ×1000 Safranin (**A**) and Geimsa (**B**) staining.

Erythrocytes of the FFC-dosed *O. niloticus* showed morphological disparities, and these are portrayed in Figure 4A,B. Striking morphological alterations were observed in the 1× group compared to the control. An increased prevalence of vacuolated and damaged erythrocytes was observed. Cellular lobopodial projections and abnormalities in nuclear morphology (notches) were commonly noted (Figure 4(Ac,d,e)). The occurrence of lymphocytes and monocytes were predominant in the 1× group alongside vacuolated and tear-drop-shaped erythrocytes (Figure 4(Bc,d,e)). Erythrocytes with an increased nucleus-to-cytosol ratio, immature erythrocytes, and smudge cells were seen in all the treatment groups. The main deviant changes in erythrocytes were tear-shaped, spindle-shaped, and degenerative erythrocytes. In the 3× group, the erythrocytes with membrane deformities, darkly stained chromatin, and cell membrane rupture were prominent (Figure 4(Af,g,h)). Furthermore, binucleated erythrocytes, karyolytic cells, nuclear blebs, and micronuclei were documented (Figure 4(Bf,g,h)). The tear-shaped and damaged erythrocytes increased on day 10 FD and returned almost to normal on day 21 PFD in the 1× group. However, the nuclei were at the extreme periphery and some erythrocytes had blebbed and notched nuclei (Figure 4(Ai,j)). Hypochromic and irregular-shaped cells were also observed on day 21 PFD in the 1× group (Figure 4(Bi,j)). The 3× group showed the persistence of membrane and nuclear deformities on day 21 PFD (Figure 4(Ak,l,Bk)), as well as micronuclei (Figure 4Bl). Although the blood cell morphological changes were higher in the dosing groups during the FD regimen, the day 21 PFD blood samples showed almost normal erythrocytic and leukocytic morphology. The cells with peripheral nuclei, membrane deformities, and micronuclei were still prominent after 3 weeks of the cessation of dosing, along with an increased number of lymphocytes.

### 3.5. Histopathology

**Figure 5:**
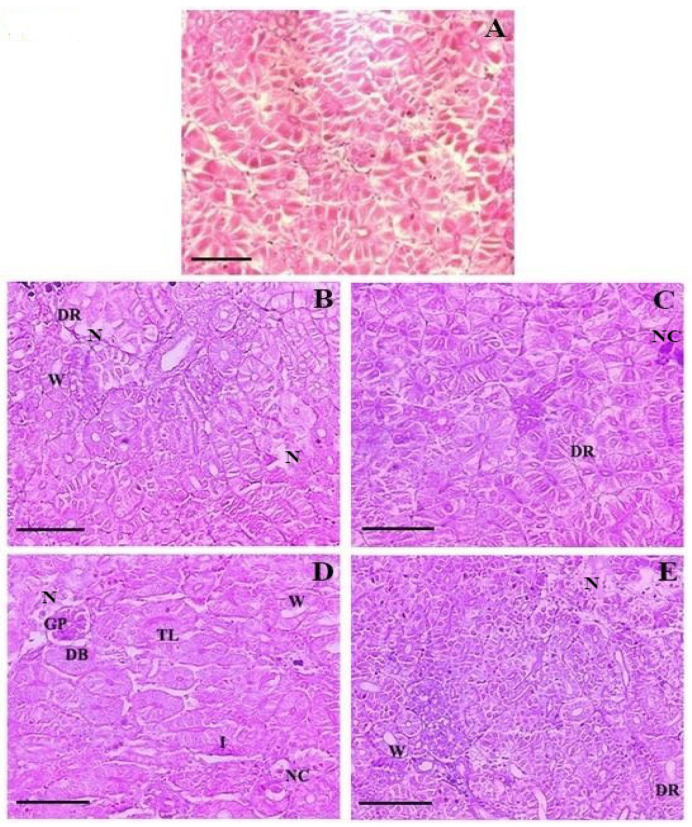
(**A**) Histological features of the kidneys of control-fed *Oreochromis niloticus* juveniles showing normal tissue architecture, ×200 H&E staining. Histopathological changes in the kidneys of florfenicol (FFC)-dosed *O. niloticus* juveniles at 15 mg kg biomass^−1^ day^−1^ (1× group) for 10 consecutive days (**B**) on day 10 FFC-dosing showing degeneration of the renal tubular epithelium (DR), necrotized renal tubules (N), and widened lumen (W), ×200 H&E staining; and (**C**) on day 21 post-FFC-dosing showing degeneration of the renal tubular epithelium (DR) and nephrocalcinosis (NC), ×200 H&E staining. Histopathological changes in the kidneys of FFC-dosed *O. niloticus* juveniles at 45 mg kg biomass^−1^ day^−1^ (3× group) for 10 consecutive days (**D**) on day 10 of FFC dosing showing glomerulopathy (GP), dilated Bowman’s space (DB), necrotized renal tubule (N), thickening of the lumen lining (TL), widened lumen (W), inflamed renal tubule (I). and nephrocalcinosis (NC), ×200 H&E staining; and (**E**) on day 21 post-FFC-dosing showing degeneration of the renal tubular epithelium (DR), necrotized renal tubule (N) and widened lumen (W), ×200 H&E staining.

The qualitative assessment of major histopathological alterations in the kidney and liver tissues of FFC-dosed *O. niloticus* juveniles is displayed in Table 4. The histopathological alterations in the kidney of FFC-dosed *O. niloticus* for 10 consecutive days are shown in Figure 5A–E. Compared to controls (Figure 5A), the 1× group displayed mild degeneration of the renal tubular epithelium and a widened lumen on day 10 FD (Figure 5B). Glomerulopathy and nephrocalcinosis were considered normal, with <5% of tissues affected. However, on day 21 of PFD, the persistence of degeneration of the renal tubular epithelium was noted in the 1× group (Figure 5C). The changes were considered normal, except for mild nephrocalcinosis. A shrunken and fragmented glomerulus with a dilated Bowman’s space, a widened lumen, inflammation of renal tubules, and nephrocalcinosis were observed to be mild in the 3× group on day 10 FD (Figure 5D). Thickening of the lumen was considered normal. On day 21 of PFD, the necrotized renal tubule, glomerulopathy, and nephrocalcinosis showed mild persistence (Figure 5E). The extent of kidney tissue damage was, however, reduced during the PFD period, with most of the adversities reaching normalcy.

**Figure 6:**
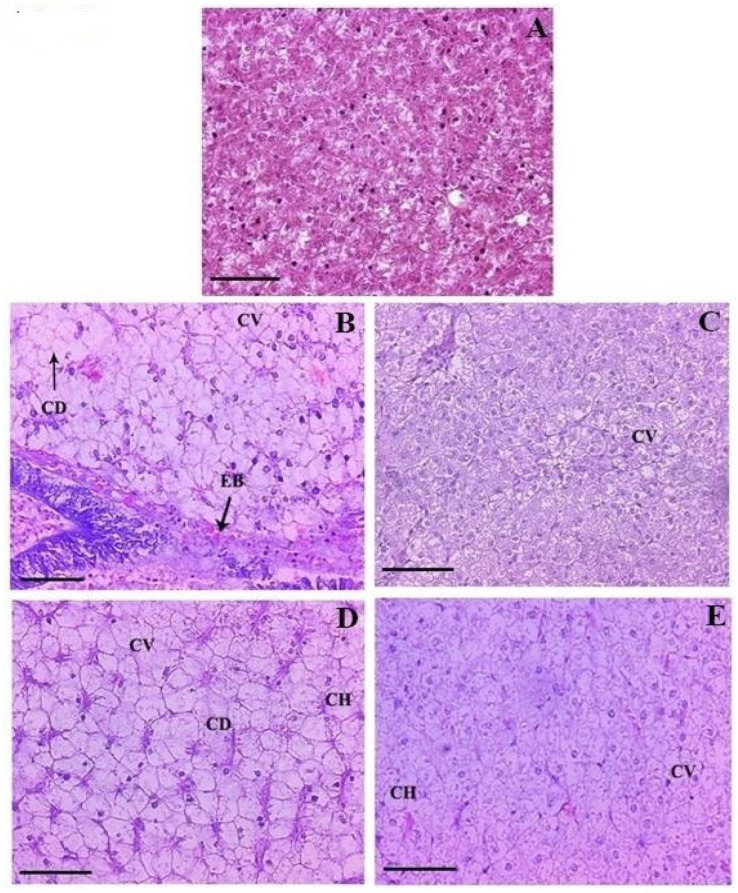
(**A**) Histological features of the livers of control-fed *Oreochromis niloticus* juveniles showing normal tissue architecture, ×200 H&E staining. Histopathological changes in the livers of florfenicol (FFC)-dosed *O. niloticus* juveniles at 15 mg kg biomass^−1^ day^−1^ (1× group) for 10 consecutive days (**B**) on day 10 of FFC dosing showing marked glycogen-type vacuolation, cytoplasmic degeneration (CD), cytoplasmic vacuolation (CV), and eosinophilic bodies (EB), ×200 H&E staining; and (**C**) on day 21 post-FFC-dosing showing mild glycogen-type vacuolation and cytoplasmic vacuolation (CV), ×200 H&E staining. Histopathological changes in the livers of FFC-dosed *O. niloticus* juveniles at 45 mg kg biomass^−1^ day^−1^ (3× group) for 10 consecutive days (**D**) on day 10 of FFC dosing showing marked glycogen-type vacuolation, cytoplasmic degeneration (CD), cytoplasmic vacuolation (CV), and cellular hypertrophy (CH), ×200 H&E staining; and (**E**) on day 21 post-FFC dosing showing marked glycogen-type vacuolation, cytoplasmic vacuolation (CV), and cellular hypertrophy (CH), ×200 H&E staining.

The liver sections of the 10-day FFC-dosed groups lost their characteristic liver architecture with markedly increased glycogen-type vacuolation in hepatocytes (Figure 6A–E). Severe glycogen-type vacuolation was noted on day 10 FD in the 1× (Figure 6B) and 3× (Figure 6D) groups, respectively. Moreover, mild cytoplasmic vacuolation, cytoplasmic degeneration, and cellular hypertrophy were noted in both groups. Eosinophilic bodies were also documented in the 1× group on day 10 FD. On day 21 FD, the intensity of damage was reduced but the persistence of cytoplasmic vacuolation in the 1× (Figure 6C), as well as considerable cytoplasmic vacuolation and cellular hypertrophy, was observed in the 3× groups (Figure 6E). Moderate persistence of glycogen-type vacuolation was noted in the 3× group on day 21 PFD.

## 4. Discussion

The 1× and 3× groups’ feed intake was significantly more abated than that of the control group, in a dose-dependent fashion, during the FD tenure, indicating the reduced acceptability of FFC feeds. Both groups showed a reduced biomass increment of 1.04–1.08-fold of their original biomass compared to the control group, indicating an impediment in fish growth during the FD periods. With the termination of dosing, the juveniles showed no abnormalities and increased interest in feeding, though insignificantly, similarly to a previous study [24]. No mortalities were noted in the 1× group, as was also observed in earlier studies at therapeutic or lower FFC dose levels [6,25,26]. At 10 days of dosing we observed significantly increased glucose levels in both groups, indicating that FFC prompted stress even at the therapeutic dose and gratified the extra demands of fish for metabolic energy under stressful conditions, similarly to the results of prior studies [6,26,27]. In the 1× group, the levels remained constant until day 14 PFD, signifying the persistence of stress. The serum calcium levels were significantly reduced on day 10 of FD, indicating a discrepancy in osmolarity and ionic balance, possibly due to FFC. FFC had a greater impact on calcium than on chloride ions. The relationship between serum calcium and FFC is uncertain. Calcium is believed to play an important role in regulating mitochondrial function. FFC-induced mitochondrial dysfunction has been demonstrated previously [28]; therefore, an inverse relationship between FFC intake and serum calcium may be predicted. Furthermore, the drug may alter the membrane permeability, contributing to a lessened intake of ionic compounds into the body, thus creating intra- and extracellular fluid imbalance. Antibiotics such as tetracyclines tend to bind to calcium, reducing its availability in serum [29]. Our observation of the increased incidence of ruptured cell membranes in the 3× group support the results of an earlier study [30], possibly due to the attachment of FFC on the erythrocytic membrane and interruption of the membrane transport system. The results of our study also indicate that freely available chlorine readily combines with FFC and transforms it for easy elimination.

Creatinine levels increased significantly in a dose-dependent manner, indicating physical-exertion- and FFC-induced renal disorder, similarly to oxytetracycline (OTC) in *O. niloticus* [27]. Nevertheless, the creatinine levels of the 1× group were recouped, indicating the reversible nature of FFC-induced stress on kidney functioning at the therapeutic dose. In contrast, the creatinine levels observed during the PFD period with the higher FFC dose suggest only a slight improvement in the renal functions of *O. niloticus* and corroborate our earlier report [6]. The serum ALT and AST levels were negatively influenced by dietary FFC in a dose-dependent manner, thus signifying liver tissue deterioration or injury. The significant increase in AST indicates that FFC at the therapeutic dose may impair liver tissues, as was observed in earlier studies [6,27]. Our results endorse the findings of another previous study [31], which documented an increase in AST upon the application of FFC in *Oncorhynchus mykiss*. The induced liver damage was further confirmed by the increased ALP observed in the 1× and 3× groups on day 10 FD and day 1 PFD, probably due to the accumulation of FFC residues in the liver tissues upon dosing. Though the exact mechanism of FFC toxicity in fish hepatocytes is not known, the observed excessive hepatic vacuolation probably exerted undue pressure on the hepatic membranes, leading to the leakage of hepatic exoenzymes such as ALT, AST, and ALP into the blood [32]. The ALP levels did not decrease with the cessation of dosing, suggesting persisting liver inflammation in the dosed fish. The role of oxidative damage in the liver tissues and cell necrosis in response to FFC toxicity [26] could not be ruled out.

A reduction in TECs was observed in both groups, similarly to OTC-fed *O. niloticus* [10] and *Cyprinus carpio* [12], gentamicin-injected *O. niloticus* [33], and amoxicillin-exposed *L. rohita* [11]. Decreased TECs are often accompanied by a reduction in Hb and Ht [12], as the antibiotics harm the hematopoietic organs of fish and induce anemia. Our observations indirectly indicate that the damage caused to the hematopoietic microenvironment and the decrease in the number of hematopoietic cells induced by FFC probably decreased the number of peripheral red blood cells (RBCs). Similarly, FFC reportedly inhibited cell division and proliferation and induced apoptosis in the bone marrow, spleen, and thymus, leading to hemotoxicity and immunotoxicity in mice [28]. The previous works on antibiotic impairment in fish hematology also documented similar observations [8,10,11,12,33,34]. Since TECs have gas exchange functions, their reduction suggested diminished gas exchange and oxygen transport to tissues. Our observations of the decrease in TECs also indicated impaired osmoregulation caused by FFC, which was supported by the histological observations of dosed fish. A significant decline in Hb content was observed in the FFC-dosed *O. niloticus*, which may be due to insufficient oxygen supply and anemic conditions. Hem content has a significant role in energy metabolism, alterations of which may lead to behavioral changes and lethargy. Though such responses were not documented in the current study, the ablation of Hb levels implied the inhibitory effect of FFC on the enzyme system, which is responsible for hemoglobin synthesis. The formation of hypochromic cells, as observed in our study, was probably due to the reduction in Hb content. Irregularly shaped erythrocytes and reduced TECs may decrease the Ht of an organism [11] and our findings corroborate these findings. The observed MCV values of the FFC-dosed *O. niloticus* possibly indicated hypoxic and anemic conditions created by the drug. Increased MCHC values also suggested a protective response of *O. niloticus* against FFC toxicity. The highly lipophilic properties of FFC may enable it to cross the membranes of erythrocytes and make them fragile and prone to disruption [6]. Furthermore, ionic imbalance demands excessive energy, which may affect the membranes of erythrocytes, as documented in the current study. The observed alterations in the number of erythrocytes may be due to a redeeming response in fish regarding FFC toxicity [34].

Antibiotic feeding is often accompanied by leukocytosis due to inductive stress and activation of the piscine immune system [12]. In the present study, we observed a considerable increase in the TLCs of FFC-dosed *O. niloticus* to protect against FFC toxicity, which persevered even 3 weeks after the cessation of dosing. This stimulatory effect may have occurred due to the probable increase in antioxidant levels by FFC [35]. The observed leukocytosis indicated a reaction by *O. niloticus* to FFC toxicity. The increase in TLCs was accompanied by a significant increase in lymphocytes and ablation in monocytes, which corroborate the findings of an earlier study [36] in mice upon FFC administration. Induced lymphocytosis, as observed in our study, is likely due to FFC toxicity, which is often preceded by inflammatory conditions or suppressed immunity in fish [3]. Thrombocytosis (excessive platelet counts) is uncommon in fish upon antibiotic exposure. It is more likely a secondary condition, observed in connection with stress. However, earlier studies reported a significant hike in the TCs of OTC-dosed *O. niloticus* [37,38]. The hike in TCs consolidates the role of thrombocytes in *O. niloticus* by transferring essential information to leukocytes, compensating for the subdued immune response upon dosing. During hypoxic conditions, the CO_2_ level may increase and the blood may become acidic, leading to the rupture of erythrocytes [9]. The observed rupture of erythrocytes, together with several lobopodial and cytosolic projections, might have altered the MCV levels in *O. niloticus*. Furthermore, an increase in MCH values is an indication of an increase in the number of irregularly shaped erythrocytes in the circulation and the swelling of RBCs [23]. The reduction in MCHC values can be attributed to the loss of Hb and damaged erythrocytes [23]. The results of our study indicated mild alterations in the hematological parameters at the therapeutic dose as a non-specific immune response to FFC toxicity. Nevertheless, the levels of hematological parameters and indices, more or less, were recouped within 3 weeks, implying that FFC-induced hematological changes are reversible.

The degree to which FFC can alter the physiology of *O. niloticus* has been determined using morphological aberrations of erythrocytes. We observed various erythrocytic cellular and nuclear abnormalities, as documented in an earlier study [39]. The FFC-induced reduction in Hb content and the resultant reduced oxygen uptake allowed the erythrocytes to alter their morphology. It is assumed that such types of cellular abnormalities may endure morphological alterations in the plasma membrane, affecting surface deformability and making the erythrocytes more susceptible to bursting when crossing small capillaries. The formation of lobopodial and cytoplasmic projections in fish erythrocytes from one side demarcated the FFC toxicity. Echinocytes are the most common shape-shifted erythrocytes, which contain several cytoplasmic projections to account for reduced Hb and hypoxic conditions. The predominant increase in lymphocytes in the dosing groups suggested FFC toxicity, stress, and lymphoid cell proliferation [40]. Although there was no tangible substantiation of hematopoietic or lymphopoietic tissue degradation, the significant increase in lymphocytes implied the stress that the fish underwent. RBCs, when exposed to certain stimuli, produce apparent morphological and molecular alterations called “shape-shifted RBCs (shRBCs)” [6,41]. In our study, several alterations in erythrocytic shape, viz., tear-drop, spindle, and longitudinally flattened shapes, were documented, which further established the cytotoxic and nucleotoxic potential of FFC, similarly to earlier studies [6,13]. The observation of the increased number of damaged erythrocytes can be attributed to FFC-induced toxicity. The increased frequency of ruptured cell membranes, particularly at the 3× dose, is an indication of the disruption of the lipid solubility of the membranes of erythrocytes. A relatively large number of smudge cells were documented in our study, which indicated lymphocytosis. Erythrocytic morphological alterations often change the intracellular Hb content [42] and our results in relation to Hb supported their findings. The increased erythrocyte malformation may be related to increased lipid peroxidation in fish tissues and oxidative stress, as was observed in earlier studies [26,43]. The presence of more cytosol projections could be attributed to the lipid membrane’s greater permeability and flexibility as a result of this accelerated peroxidation.

The malformation of erythrocytic nuclei suggested the nucleotoxic effects of FFC, which can cause DNA damage by changing the DNA base and interrupting DNA strands. The notched nuclei of the altered erythrocytes that were revealed in the FFC-dosed *O. niloticus* may have blistered. The increased frequency of nuclear notches and blebs may shed further light on the nucleotoxic properties of FFC. Karyolysis, peripheral nuclei, reduced hypochromic cytosol, and vacuolations were also observed at the therapeutic dose. An increase in the frequency of micronuclei in erythrocytes indicated FFC-induced chromosomal abnormalities and a disturbance in the mitotic process of *O. niloticus*. Regardless of the underlying causes, an increase in micronuclei may raise the risk of genetic and degenerative problems due to unstable chromosomes and damaged DNA. Dark-stained nuclear chromatin was observed in many erythrocytes of the 3× group on day 10 FD. The significantly increased percentile rate of erythrocytes, along with the notched nuclei, micronuclei and blebbed nuclei, possibly indicated a higher production of caspase-activated DNase and oxidative stress to mitochondria, causing the breakage of cytoskeletal and nuclear proteins [44]. Nevertheless, the therapeutic dose did not show any signs of cell rupture, indicating the safety of FFC. Upon the cessation of dosing, the blood smears revealed the increased prominence of mature erythrocytes and healthier cellular elements in the therapeutic group. The elimination of stress also terminated the formation of shRBCs and reversed lymphocytes’ increased prominence. However, nuclear abnormalities were still observed in all the treatment groups, indicating that perhaps the fish may have required more time to recover. Though FFC-induced cytotoxicity and nucleotoxicity were reversible, their impact on genotoxicity requires further study.

Histopathological investigations have long been accepted to be reliable biomarkers of stress in fish [45]. Mild-to-moderate histological alterations were observed in the kidneys, probably indicating the nephrotoxic effect of FFC upon oral dosing at 15–45 mg kg biomass^−1^ day^−1^, with an increased intensity of damage at the higher dose. Likewise, reports of mild tubular degeneration in *O. niloticus* upon FFC administration at 15 mg kg biomass^−1^ day^−1^ are available [40]. The presence of tubule degeneration, coupled with the absence of necrosis in the kidneys of *O. niloticus*, in the present study indicated that the kidneys suffered damage upon dosing, but the lower dose may have barred the development of necrosis in this organ. Furthermore, when the kidney is stimulated by FFC, a large number of ROS radicals and apoptosis-related factors are produced, which attack the cell membranes of nephrons and aggravate the degree of renal injury and cell damage [46]. The observed excessive enlargement of the tubular epithelium in our study provided evidence for renal injury. Glomerulopathy with a dilated Bowman’s space is an indication of the defective glomerular filtration of blood and defective removal of excess wastes and fluids. The observations related to inflammation and the loss of structural integrity of the renal tubules in FFC-dosed fish suggested a prolonged excretion time. Nephrocalcinosis (mineralization) was observed in this study on day 10 FD in the 3× group, corroborating a previous work [40], possibly due to the elevated ambient CO_2_ levels and mineral imbalances. The observed histopathological changes in the kidneys can also be correlated with elevated serum creatinine levels and impaired kidney functions, particularly the glomerular filtration rate. Furthermore, the renal toxicity of FFC was positively correlated with its dose. With the termination of dosing, the FFC-induced renal injury was alleviated, as confirmed by the serum creatinine levels.

There was a dose-related increased incidence of mild-to-marked hepatocyte vacuolation in FFC-dosed *O. niloticus*. Glycogen depletion in the hepatocytes is usually found in antibiotic-stressed fish [47] because the glycogen acts as a reserve of glucose to supply the higher energetic demand occurring in such situations [48]. The observation of excessive glycogen-type vacuolation in the liver could be attributed to glycogenesis. There is compelling evidence that excess stress might lead to hepatic disorders, which greatly reduce the food consumption and growth performance of fish [6,26,27], similarly to the findings of the present study. Among several metabolic disturbances, glycogenic hepatopathy (hepatomegaly) and cytoplasmic vacuolation were the most common ones observed in our study. The well-endorsed ability of *O. niloticus* to maintain glucose homeostasis under stress conditions is largely unknown. The dose-dependent significant decrease in food consumption, especially during the latter part of the dosing period in both groups, may have affected the energy balance [49], and thus possibly led to hepatocyte vacuolation in FFC-dosed fish. Dietary FFC induced the production of eosinophilic bodies, possibly associated with chronic inflammatory reactions in the liver tissues, as the inflammation was more pronounced in the liver. The documentation of eosinophilic bodies in the 1× group is indicative of apoptosis and is often accompanied by swelling of the hepatocytes [50]. A dose-dependent varying degree of cytoplasmic degeneration was documented in FFC-dosed groups, suggesting the depletion of the glycogen reserves in the hepatocytes and stress on fish. The magnitude of the increase in AST, ALT, and ALP levels crudely reflected the number of affected hepatocytes. Although the intensity of vacuolation recovered slightly, it was still observed on day 21 of PFD. The overall toxic impact of FFC on the kidneys and liver may seriously affect metabolic as well as physiologic activities and impair the growth of fish.

The application of FFC has led to increased interest in its toxic and side effects, including its hematopoietic toxicity, nucleotoxicity, immunotoxicity, genotoxicity, and embryonic toxicity in animals [51]. Our observations of hypochromic erythrocytes and drastic reductions in erythrocytes, Hb content, and other hematological indices suggest the induction of adverse effects in FFC high-dose therapy. These results further suggest the interference of FFC with the heme synthesis and hematopoietic tissue, similarly to amphenicol antimicrobials in mammals, but the hematopoietic suppression was reversible [52]. The decline in Hb is also related to the decreased erythrocytic population. High-dose therapy may produce adverse effects on major tissues and organs and interfere with various pathways in hematopoietic toxicity, such as cytolysis, genotoxicity, and the inhibition of mitochondrial protein synthesis [52]. Previous studies indicated that FFC contaminants in the environment can enter the human body through the food chain and water, with results displaying marked mitonuclear protein imbalances and microbiome disturbances [46,53,54]; therefore, our study calls for the responsible use of FFC for bacterial disease control in aquaculture.

## 5. Conclusions

Dietary FFC influenced the biological and physiological state of *O. niloticus* juveniles in a dose-dependent manner. The results of this study showed a significant decrease in feed consumption at the therapeutic dose, which recovered within 3 weeks after the cessation of dosing. No mortality was observed at the therapeutic dose and duration. However, the higher FFC dose caused significant aberrations and alterations in the piscine body and deferred the recovery. Though there were anomalies in serum biomarker levels, blood cell and nuclear morphologies, and hematological parameters, these variances were reversible with the suspension of dosing. Our results thus suggest that dietary FFC is well tolerated by *O. niloticus* and support the claim that it is safe at the therapeutic level. This study thus supports the safety of FFC at the maximum therapeutic dose of 15 mg kg biomass^−1^ day^−1^ for 10 consecutive days in *O. niloticus* under tropical Indian conditions.

## Figures and Tables

**Table 1:** The details on the serum biomarkers tested and kits used to assess the effect of dietary florfenicol in *Oreochromis niloticus*.

Serum Biomarkers	Kits Used	Reference
Glucose	Glucose test kit, GOD FS 10′ (Diasys Diagnostic Systems, Germany)	[15]
Calcium	Calcium test kit, AS FS (Diasys Diagnostic Systems, Germany)	[16]
Chloride	Chloride test kit, 21 FS (Diasys Diagnostic Systems, Germany)	[17]
Creatinine	Creatinine test kit, Modified Jaffe’s Reaction, Initial rate assay (Span Diagnostics Ltd., India)	[18]
ALT ^1^	ERBA SGPT Kit, IFCC Method, Kinetic (Erba Manheim, Germany)	[19]
AST ^2^	ERBA SGOT Kit, IFCC Method, Kinetic (Erba Manheim, Germany)	[19]
ALP ^3^	Alkaline Phosphatase Kit, FS IFCC 37 °C (Diasys Diagnostic Systems, Germany)	[20]

^1^ ALT: alanine aminotransferase, ^2^ AST: aspartate aminotransferase, ^3^ ALP: alkaline phosphatase.

**Table 2:** Visual characterization of behavioral abnormalities in *Oreochromis niloticus* juveniles upon dietary administration of florfenicol at 0–3 times the therapeutic dose (**1**×: 15 mg kg biomass^−1^ day^−1^) for 10 consecutive days in comparison to the control.

Behavioral Changes ^a^	Percentage of Fish					
	Day 10 FD			Day 21 PFD		
	0×	1×	3×	0×	1×	3×
Gasping for air	0.00 (0)	7.50 (9)	11.67 (14)	0.00 (0)	2.50 (3)	4.17 (5)
Lethargy	0.00 (0)	0.00 (0)	1.67 (2)	0.00 (0)	0.00 (0)	0.00 (0)
Excessive epidermal mucus secretion	0.00 (0)	4.17 (5)	10.83 (13)	0.00 (0)	0.00 (0)	0.83 (1)
Residing at tank bottom *	0.00 (0)	8.33 (10)	9.17 (11)	0.00 (0)	0.00 (0)	0.00 (0)

^a^ Characteristics were evaluated on stocked fish for each treatment (n = 120) and the pooled data are presented. * The rest of the fish were observed to be distributed throughout the water column. Values in parentheses indicate the total number of fish that demonstrated the particular abnormality. FD: florfenicol dosing; PFD: post-florfenicol dosing.

**Table 3:** Details of the hematological biomarkers tested and the kits used to assess the effect of dietary florfenicol in *Oreochromis niloticus*.

Biomarkers	PD	Day 10 FD			Day 21 PFD		
		0×	1×	3×	0×	1×	3×
TEC (×10^6^ cells mm^−3^)	1.73 ± 0.06 *	1.70 ± 0.06 ^1a^ *	1.19 ± 0.03 ^1b^	1.14 ± 0.06 ^1b^	1.73 ± 0.05 ^1a^ *	1.36 ± 0.05 ^2b^	1.26 ± 0.04 ^2c^
TC (×10^5^ cells mm^−3^)	1.26 ± 0.12 *	1.28 ± 0.03 ^1a^ *	1.89 ± 0.02 ^1b^	1.96 ± 0.06 ^1c^	1.26 ± 0.02 ^1a^ *	1.31 ± 0.01 ^2a^	1.37 ± 0.02 ^2a^
TLC (×10^4^ cells mm^−3^)	3.09 ± 0.05 *	3.08 ± 0.03 ^1a^ *	10.33 ± 0.05 ^1b^	11.85 ± 0.04 ^1c^	3.09 ± 0.06 ^1a^ *	9.13 ± 0.05 ^2b^	9.41 ± 0.10 ^2b^
LC (%)	65.36 ± 0.22 *	65.58 ± 1.20 ^1a^ *	86.57 ± 1.46 ^1b^	88.88 ± 0.59 ^1c^	64.95 ± 1.19 ^1a^ *	73.32 ± 1.00 ^2b^	78.20 ± 1.39 ^2c^
MC (%)	4.53 ± 0.38 *	4.54 ± 0.32 ^1a^ *	2.23 ± 0.15 ^1b^	1.13 ± 0.06 ^1c^	4.57 ± 0.33 ^1a^ *	3.87 ± 0.06 ^2b^	2.70 ± 0.53 ^1c^
Hb (g dL^−1^)	4.97 ± 0.32 *	4.97 ± 0.21 ^1a^ *	4.20 ± 0.17 ^1b^	3.53 ± 0.25 ^1c^	5.01 ± 0.25 ^1a^ *	4.64 ± 0.35 ^2b^	3.77 ± 0.15 ^1c^
Ht (%)	19.09 ± 0.33 *	19.12 ± 0.65 ^1a^ *	18.25 ± 0.79 ^1a^	16.41 ± 0.44 ^1b^	19.53 ± 0.31 ^1a^ *	18.62 ± 0.53 ^1a^	17.60 ± 0.14 ^2b^
MCV (10^−15^ L)	112.79 ± 0.99 *	112.78 ± 2.50 ^1a^ *	143.41 ± 9.93 ^1b^	153.48 ± 9.04 ^1c^	113.01 ± 5.17 ^1a^ *	137.25 ± 1.12 ^2b^	140.13 ± 4.04 ^2c^
MCH (10^−12^ g)	28.81 ± 1.59 *	29.03 ± 1.29 ^1a^ *	30.95 ± 2.06 ^1a^	35.32 ± 3.90 ^1b^	29.13 ± 2.13 ^1a^ *	29.98 ± 2.29 ^1a^	32.44 ± 1.76 ^2b^
MCHC (g dL^−1^)	25.13 ± 1.62 *	25.78 ± 0.19 ^1a^ *	21.11 ± 2.51 ^1b^	20.82 ± 1.69 ^1b^	25.48 ± 1.69 ^1a^ *	22.02 ± 2.25 ^2b^	21.84 ± 1.75 ^2b^

Values are expressed as the mean ± standard deviation. Values of the parameters observed during the pre-dosing period differed significantly (*p* < 0.05) among all the FFC-dosed groups. PD: pre-dosing period; FD: FFC-dosing; PFD: post-FFC-dosing; TEC: total erythrocyte count; TLC: total leukocyte count; TC: thrombocyte counts; LC: lymphocyte counts; MC: monocyte counts; Hb: hemoglobin; Ht: hematocrit; MCV: mean corpuscular volume; MCH: mean corpuscular hemoglobin; MCHC: mean corpuscular hemoglobin concentration. 1–2: values sharing common numeral superscripts for a particular treatment within a particular row differed insignificantly (*p* > 0.05). a–c: values sharing common alphabetical superscripts for a particular dosing period within a particular row differed insignificantly (*p* > 0.05). *: values sharing asterisks (*) within a particular row differed insignificantly (*p* > 0.05).

**Table 4:** Qualitative assessment of major histopathological changes in the kidney and liver tissues of florfenicol-dosed Nile tilapia *Oreochromis niloticus* juveniles at 0–3 times the therapeutic dose of 15 mg kg biomass^−1^ day^−1^ for 10 consecutive days on a 5-point ordinal scale * in comparison with the normal architecture.

Major Histopathological Changes	1×		3×	
	Day 10 FD	Day 21 PFD	Day 10 FD	Day 21 PFD
**Kidney**				
Degeneration of renal tubular epithelium	1.38 ± 0.11 ^1a^	0.48 ± 0.24 ^2a^	1.54 ± 0.10 ^1b^	0.97 ± 0.10 ^2b^
Necrotized renal tubule	1.18 ± 0.11 ^1a^	0.37 ± 0.21 ^2a^	1.28 ± 0.10 ^1a^	1.14 ± 0.10 ^2b^
Glomerulopathy with dilated Bowman’s space	0.43 ± 0.15 ^1a^	0.19 ± 0.07 ^2a^	1.17 ± 0.10 ^1b^	1.05 ± 0.13 ^2b^
Nephrocalcinosis	0.78 ± 0.28 ^1a^	1.02 ± 0.13 ^2a^	1.21 ± 0.07 ^1b^	1.11 ± 0.07 ^2a^
Thickening of lumen lining	0.00 ± 0.00 ^1a^	0.00 ± 0.00 ^1a^	0.43 ± 0.08 ^1b^	0.00 ± 0.00 ^1a^
Inflamed renal tubule	0.00 ± 0.00 ^1a^	0.00 ± 0.00 ^1a^	1.11 ± 0.15 ^1b^	0.62 ± 0.34 ^2b^
Widening of lumen	1.23 ± 0.13 ^1a^	0.82 ± 0.27 ^2a^	1.13 ± 0.14 ^1b^	0.52 ± 0.16 ^2b^
**Liver**				
Glycogen type vacuolation	4.59 ± 0.05 ^1a^	1.46 ± 0.04 ^2a^	4.67 ± 0.02 ^1b^	2.54 ± 0.04 ^2b^
Cytoplasmic vacuolation	1.31 ± 0.07 ^1a^	1.27 ± 0.10 ^1a^	1.42 ± 0.07 ^1b^	1.28 ± 0.13 ^2a^
Cytoplasmic degeneration	1.04 ± 0.14 ^1a^	1.00 ± 0.10 ^1a^	1.11 ± 0.09 ^1a^	1.09 ± 0.07 ^1a^
Cellular hypertrophy	1.01 ± 0.07 ^1a^	0.86 ± 0.19 ^2a^	1.44 ± 0.10 ^1b^	1.08 ± 0.07 ^2b^

* Qualitative assessment ordinal scale: 0 = no change; 1 = normal with <5% of tissues affected; 2 = mild with 5–15% of tissues affected; 3 = moderate with 15–25% of tissues affected; 4 = marked with 25–50% of tissues affected and 5 = severe with >50% of tissues affected. The qualitative assessment was based on six observations (mean ± standard deviation) for each organ of the respective group. No changes were noted in the control group. FD: florfenicol dosing; PFD: post-florfenicol dosing. 1, 2: values sharing common numeral superscripts in a particular row for a particular treatment differed insignificantly (*p* > 0.05). a, b: values sharing common alphabetical superscripts in a particular row for a particular day differed insignificantly (*p* > 0.05).

## Data Availability

The data presented in this study are available on request from the corresponding author.
